# Evaluating the Diagnostic Performance of Systemic Immune-Inflammation Index in Childhood Inflammatory Arthritis: A Focus on Differentiating Juvenile Idiopathic Arthritis from Reactive Arthritis

**DOI:** 10.3390/biomedicines12010065

**Published:** 2023-12-27

**Authors:** Delia-Maria Nicoară, Andrei-Ioan Munteanu, Alexandra-Cristina Scutca, Giorgiana-Flavia Brad, Raluca Asproniu, Iulius Jugănaru, Otilia Mărginean

**Affiliations:** 1 Department XI Pediatrics, Discipline I Pediatrics, ‘Victor Babeş’ University of Medicine and Pharmacy of Timisoara, 300041 Timisoara, Romania; nicoara.delia@umft.ro (D.-M.N.); scutca.alexandra@umft.ro (A.-C.S.); brad.giorgiana@umft.ro (G.-F.B.); raluca_asproniu@yahoo.com (R.A.); juganaru.iulius@umft.ro (I.J.); marginean.otilia@umft.ro (O.M.); 2 Department of Pediatrics I, Children’s Emergency Hospital “Louis Turcanu”, 300011 Timisoara, Romania; 3 Research Center for Disturbances of Growth and Development in Children BELIVE, ‘Victor Babeş’ University of Medicine and Pharmacy of Timisoara, 300041 Timisoara, Romania

**Keywords:** juvenile idiopathic arthritis, reactive arthritis, inflammation, complete blood count, systemic immune-inflammation index

## Abstract

In pediatric care, the range of potential diagnoses for arthritis can be relatively extensive, primarily involving infectious and inflammatory causes and, to a lesser extent, oncological conditions. Specifically, when addressing inflammatory causes, differentiating between Juvenile Idiopathic Arthritis (JIA) and Reactive Arthritis (ReA) can prove to be challenging during the first weeks, owing to the lack of specific antibodies in several JIA subtypes. This single-center retrospective study of 108 children with arthritis aimed to evaluate in greater detail the complete blood count (CBC) profiles of children with JIA and ReA in greater detail. The most significant differences were noted in terms of the Systemic Immune-Inflammation Index (SII), with higher values in the JIA group. Moreover, within the JIA group, SII displayed a significant positive correlation with conventional inflammatory biomarkers, specifically C-reactive protein (ρ = 0.579) and Erythrocyte Sedimentation Rate (ρ = 0.430). It was the only independent factor associated with the presence of JIA after adjusting for age (*p* = 0.030). Also, even with the moderate diagnostic value, the discriminating capacity of SII was superior to those of each of its component CBC parameters according to receiver operating characteristic (ROC) analysis. In summary, this study identified elevated SII values in the JIA group compared to the ReA group, indicating the potential utility of SII as an adjuvant discriminatory marker between these two arthritis forms.

## 1. Introduction

Juvenile idiopathic arthritis (JIA), the most frequent childhood rheumatic disease, has a prevalence of up to 4 per 1000 children and adolescents [1,2]. Rather than a single disorder, JIA encompasses various patient categories determined by the type of joint involvement and the presence/absence of particular serologic markers [3]. However, these specific markers can be demonstrated in only a portion of JIA subtypes, such as anti-cyclic citrullinated peptide antibodies and rheumatoid factor (RF) in polyarticular RF-positive JIA [4,5,6,7] and the human leukocyte antigen B27 in most patients with Enthesitis-Related Arthritis [8]. In some cases, there is no specific diagnostic biomarker, constituting a real challenge during the first weeks of evolution [9]. This is particularly the case when the clinical picture resembles that of Reactive Arthritis (ReA), another inflammatory joint disorder often induced by a bacterial infection in an extra-articular site [10]. It is essential to ensure rapid and accurate diagnosis, as management and long-term outcomes can be significantly different between the two types of arthritis [11]. As such, there is a growing demand for dependable biomarkers that can assist healthcare providers in differentiating JIA from other forms of arthritis, enabling timely intervention and improving patient outcomes [12,13]. During recent years, researchers have explored several complete blood count (CBC)-derived inflammation indices to enhance the diagnostic accuracy of different inflammatory disorders, given their low cost and availability [14,15,16,17,18,19,20,21]. In addition to more investigated indices, such as the neutrophil-to-lymphocyte ratio (NLR), an emerging marker is the Systemic Immune-Inflammation Index (SII), a composite score that integrates the three main peripheral blood parameters involved in inflammation, neutrophil, lymphocyte, and platelet counts [22,23,24,25]. This composite inflammation marker, which integrates three blood cell lineages into a singular parameter [26], has been studied in various autoimmune diseases, among which are Rheumatoid Arthritis [27], Ankylosing Spondylitis [28], Behcet’s Disease [29] and Antineutrophil Cytoplasmatic Antibody-Associated Vasculitis [30]. However, to the best of our knowledge, there is no information regarding the potential value of SII in Juvenile Idiopathic Arthritis (JIA). 

Our aim was to evaluate the variations in complete blood count parameters between children with active JIA and those with Reactive Arthritis, emphasizing the diagnostic performance of SII in discriminating between children with these two types of arthritis.

## 2. Materials and Methods

### 2.1. Study Design and Patient Selection

**Figure 1:**
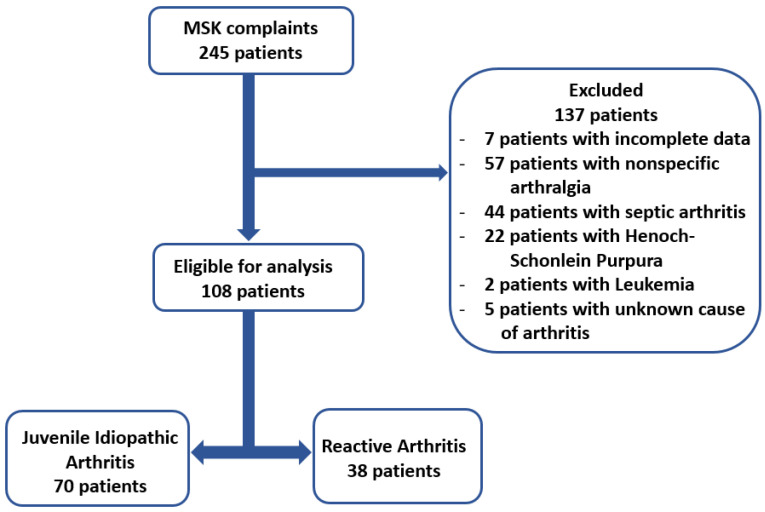
Flow chart illustrating the process for selecting patients.

This retrospective cross-sectional study was conducted in a tertiary referral center for pediatric rheumatology. We reviewed the medical records of 245 consecutive patients admitted to the Pediatric Emergency Hospital “Louis Turcanu” from Timisoara, Romania, between January 2015 and July 2023 for musculoskeletal complaints. The inclusion criteria were (1) age under 18 years and (2) diagnosis of arthritis or arthralgia. Exclusion criteria were (1) septic arthritis or Lyme arthritis, (2) active infections, (3) diseases known to modify hematological parameters, and (4) patients with incomplete data. Upon implementing the exclusion criteria, the study population included 108 patients, as seen in Figure 1.

These patients were divided into two study groups. The first group consisted of patients diagnosed with Juvenile Idiopathic Arthritis according to the International League Associations for Rheumatology (ILAR) classification [3]. The second group included patients diagnosed with Reactive Arthritis following an enteric or digestive infection [31] or after a streptococcal infection [32]. Depending on the number of affected joints, oligoarticular involvement was considered if less than five joints were affected, while polyarticular involvement was considered otherwise. 

This study was performed in accordance with the Declaration of Helsinki (1975, revised in 2013) and approved by the Institutional Review Board of our hospital. Informed consent was not necessary, owing to the retrospective nature of the study.

### 2.2. Collection of Clinical Data

The following patient data were extracted: demographic characteristics (age, gender), discharge diagnosis, and type of articular involvement (arthritis/arthralgia, number and type of affected joints). Blood analyses taken at the time of admission to the hospital that were analyzed in this study included a complete blood count (CBC) performed on an automated hematology analyzer (Sysmex XN-550, Sysmex Corporation, Kobe, Japan) and biochemistry tests. The latter, which included C-reactive protein (CRP), gamma globulins, and immunoglobulin G (IgG), were performed using an automatic analyzer (Hitachi 747, Hitachi, Tokyo, Japan). Fibrinogen was measured by the Clauss method using an ACL Top Analyzer. D-dimers were measured using an automated chemiluminescent assay (Cobas E 411-Roche, Tokyo, Japan). Additionally, the following two CBC-derived indices were computed based on the available complete blood count (CBC) taken upon admission: NLR (neutrophil count/lymphocyte count) and SII (platelet count × NLR).

### 2.3. Statistical Analysis

The two study groups were characterized using descriptive statistics (percentage, median, range of quarters (IQR)). Visual (histograms, probability plots) and analytical methods (Kolmogorov–Smirnov test) were used to evaluate the normality of data distribution. Numerical variables with abnormal distribution were expressed as median (25th and 75th interquartile range (IQR)) and compared using the Mann–Whitney U test. Categorical variables were presented as numbers (percentages), and a Chi-squared test was performed to compare these variables. R-values for intergroup comparisons were included as an additional effect size measure. R-values nearing 1 or −1 indicate a stronger effect size. The correlation between the two CBC-derived indices, NLR and SII, and several clinical variables was evaluated using Spearman’s rank correlation coefficient (ρ). Furthermore, the discriminatory value of the SII in identifying JIA patients was evaluated using a receiver operating characteristic (ROC) curve. Youden’s index, calculated as sensitivity + specificity − 1, was used to estimate cutoff values for different biomarkers. ROC curves were plotted for the inflammation markers to compare the discriminatory ability of the examined variables in identifying JIA patients. The area under the curve (AUC) in the ROC analysis was determined to compare the results. Binary logistic regression analysis was performed to determine the relationship between laboratory markers of inflammation (NLR, SII, CRP, ESR) and JIA. While exploring predictor variables, we identified instances where certain combinations resulted in sparse data. Therefore, we considered grouping ESR and age into broader categories. For ESR, we employed a 25 mm/h cutoff as determined by the Youden index we calculated. We used a 3-year cutoff for age, considering the distinctive pattern of WBC subsets known to be present in small children [33]. This way, we addressed sparsity concerns, increasing the number of observations in each category, thereby reducing the risk of the “empty cells” effect while preserving the clinical relevance of the variables in our analysis. Statistical analyses were conducted using Statistical Package for Social Sciences software (SPSS v28.0.1.1. Armonk, NY, USA, IBM Corp), and a *p*-value (two-tailed) < 0.05 was considered statistically significant.

## 3. Results

### 3.1. Patient Demographics and Characteristics

A total of 108 arthritis patients, with a median age of 10.6 (IQR: 5.4, 14.3) years, were included in the study. Of these, 70 were diagnosed with JIA, and 38 were diagnosed with Reactive Arthritis. Within the group of patients with JIA, the most prevalent subtypes were Enthesitis-Related Arthritis (31.4%), Oligoarthritis (27.1%), and RF-Negative Polyarthritis (20%), as depicted in Supplementary Table S1. 

There were no significant differences regarding gender distribution among the two study groups, as seen in Table 1. Children from the ReA group tended to be younger, with a median age of 7.7 (IQR: 3.5, 11.9) years, while those from the JIA group were older, with a median age of 12.1 (IQR: 7.6, 14.5) years. Oligoarticular involvement was prevalent among both study groups, with the ankle and knee being the most frequently involved joints. Regarding the biochemical parameters, children with JIA displayed significantly higher ESR levels (*p* = 0.017); CRP, gamma globulins, and IgG levels were also more elevated in the JIA group, although not reaching statistical significance (*p* = 0.093, 0.158, and 0.085, respectively). Both groups were similar concerning fibrinogen levels.

### 3.2. Comparision of Hematological Parameters and Indices across Study Groups

Both JIA and ReA groups were similar in terms of white blood cells (WBCs), hemoglobin (Hb), and red cell distribution width (RDW), as seen in Table 2. The most significant difference regarding CBC parameters was noted for platelets, which were higher in JIA patients compared to the ReA group (*p* < 0.001); also, neutrophils and monocytes were significantly higher (*p* = 0.046 and *p* = 0.025, respectively), whereas eosinophils exhibited a significant decrease (*p* = 0.049) in JIA patients. A trend toward lower lymphocyte count was also noted in JIA patients, although it did not reach statistical significance (*p* = 0.090). Regarding CBC-derived indices, both NLR and SII were higher in children with JIA than in those with Reactive Arthritis. 

### 3.3. Correlation Analysis of SII and NLR across Groups

**Figure 2:**
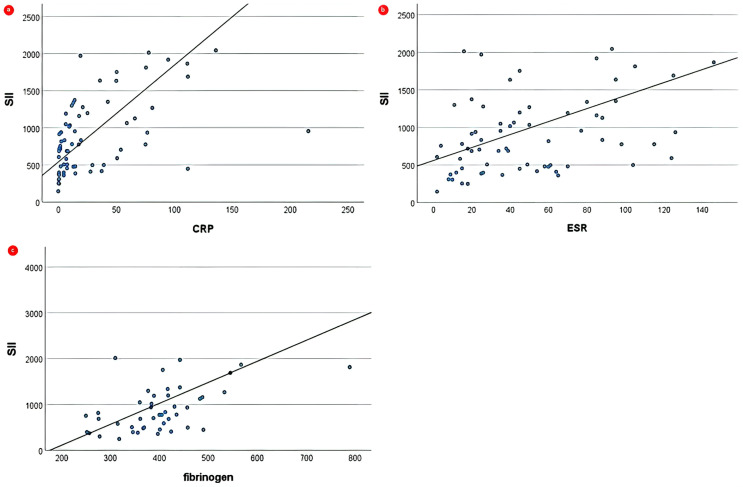
Correlations between SII and (**a**) CRP, (**b**) ESR, and (**c**) fibrinogen across JIA group. Solid lines represent linear regression lines.

Spearman correlations of SII and NLR with biochemical parameters are summarized in Supplementary Table S2. Both CBC-derived indices displayed significant positive correlations across the JIA group. NLR displayed a strong positive correlation with CRP (ρ = 0.563) and a moderate positive correlation with fibrinogen (ρ = 0.418). SII revealed a strong positive correlation with both CRP (ρ = 0.579) and fibrinogen (ρ = 0.531) and a moderate positive correlation with ESR (ρ = 0.430), as depicted in Figure 2. In the ReA group, both NLR and SII indicated significant positive correlations exclusively with CRP (ρ = 0.463 and ρ = 0.366, respectively).

### 3.4. Association between CBC-Derived Indices and JIA

We performed binary logistic regression to further characterize the relationship between the two CBC-derived indices and JIA. According to univariate logistic regression, the variables associated with the diagnosis of JIA were ESR, SII, and age (*p* = 0.036, *p* = 0.005, and *p* = 0.001, respectively). Given the relatively small sample size, we grouped ESR and age into broader categories to reduce the risk of the “empty cells” effect. As can be seen in Table 3, according to multivariate analysis, only SII retained significance as an independent factor associated with the presence of JIA after adjusting for age and ESR (*p* = 0.037).

### 3.5. Predictive Performances of Inflammatory Parameters in Discriminating JIA Patients

**Figure 3:**
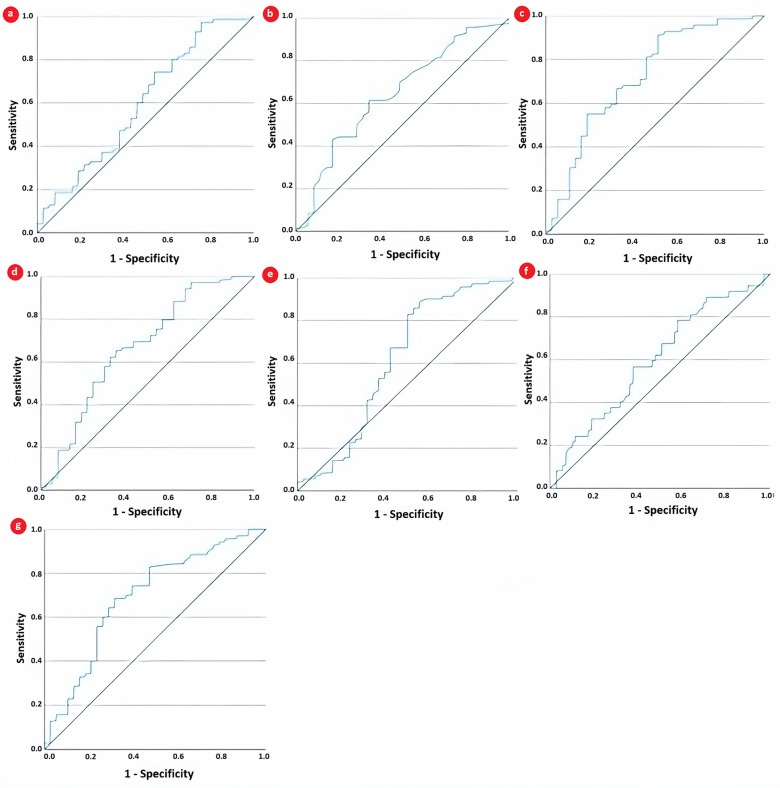
ROC curve of (**a**) CRP, (**b**) ESR, (**c**) SII, (**d**) NLR, (**e**) neutrophils, (**f**) lymphocytes, and (**g**) platelets for predicting partial clinical remission.

A receiver operating characteristic analysis was further employed to determine the diagnostic accuracy of CBC-derived indices in JIA and to compare them with the two traditional inflammatory markers, CRP and ESR. As depicted by the AUC for each parameter from Table 4 and Figure 3, SII was the only one with acceptable discrimination capacity (0.722, 95% CI: 0.615–0.829, *p* < 0.001). The AUCs obtained by NLR and ESR, 0.668 and 0.644, indicated only fair discrimination capacity, although they were statistically significant. Furthermore, the discriminating capacity of SII was superior to that of each of its component CBC parameters.

## 4. Discussion

In pediatric settings, the spectrum of potential arthritis diagnoses can be relatively broad, primarily including infectious and inflammatory causes and, to a lesser extent, oncological diseases [34,35,36,37]. With respect to inflammatory causes, distinguishing between JIA and other types of arthritis can prove to be a challenge, mainly because of the lack of specific antibodies in several JIA subtypes [35,38]. Over time, research has focused on investigating potential markers to assist clinicians in discriminating arthritis patients more accurately [13,39]. To the best of our knowledge, this is the first study to investigate the clinical utility of NLR and SII in distinguishing between JIA and Reactive Arthritis. We identified higher SII values in children with active JIA than in those with Reactive Arthritis, suggesting an auxiliary value of SII in discriminating between the two forms of arthritis.

Juvenile Idiopathic Arthritis is an immune-mediated inflammatory disease [40]. In addition to local joint inflammation [41], the ongoing inflammatory activity can alter the shape, size, and number of various cellular lineages within the hematopoietic system [42,43]. In their study, Parackova et al. described the proinflammatory function of peripheral neutrophils and their interplay with platelets in developing JIA [44]. Since the direct examination of affected tissues is limited by its invasive nature, an indirect assessment of these cells in peripheral blood could aid in characterizing the degree of inflammation [27]. In this regard, the value of CBC-derived indices in different rheumatologic diseases has been debated during the last decade, particularly in the adult age group. NLR, the most investigated CBC-derived index, has received particular focus regarding its predictive value in Rheumatoid Arthritis [45,46], Ankylosing Spondylitis [47,48], Systemic Lupus Erythematosus [49,50,51], Behçet’s Disease [52], and Sarcoidosis [53]. Moreover, NLR was positively correlated with disease activity in Rheumatoid Arthritis [49,50,51,52,53,54]. More recently, SII, a comprehensive index that integrates the three main hematological components, neutrophils, lymphocytes, and thrombocytes, has also been studied in autoimmune diseases, such as Rheumatoid Arthritis [27,55,56,57,58,59], Spondyloarthropathy [26,28,60], Psoriatic Arthritis [61,62], Behçet’s Disease [29], and Antineutrophil Cytoplasmatic Antibody-Associated Vasculitis [30]. Research on these indices in children with arthritis is notably scarce and with conflicting results [63,64]. Güneş et al. reported elevated NLR values in both active and inactive JIA patients (2.11 ± 1.19 and 2.03 ± 1.51, respectively), as opposed to healthy control subjects (1.33 ± 0.66), thus implying that the index may be of use in discriminating JIA [64]. However, when using children with other types of arthritis as a control group, Sahin et al. did not confirm the discriminatory value of NLR [65]. Neither did Li et al., who used children with Reactive Arthritis as a control group and obtained comparable NLR results between the two arthritis categories (median of 2.8, IQR: 0.3–22.0, and 3.13, IQR: 0.1–14.5, respectively) [66]. The results of our study align with those from the latter two studies. Despite active JIA patients exhibiting markedly higher NLR values compared to those with Reactive Arthritis (median of 2.12, IQR: 1.29–3.07, and 1.33, IQR: 0.84–2.19, respectively), logistic regression analysis did not establish a significant association between NLR and a JIA diagnosis. To the best of our knowledge, there are no reported studies evaluating the potential use of SII in children with JIA. Our findings revealed substantial differences in median SII between the two groups, with children in the JIA group demonstrating markedly higher values (779.9, IQR: 480.7, 1233.5) compared to those in the ReA group (410, IQR: 266, 737). Furthermore, SII showed significant correlations with both classic inflammatory markers, CRP and ESR, only in children with JIA. These two inflammatory markers have been extensively investigated in JIA [64,67]. They are incorporated by the American College of Rheumatology in the guidelines as biomarkers for JIA management [68]. ESR, in particular, is a key component of the Juvenile Arthritis Disease Activity Score, a composite disease activity score used in assessing JIA [69,70]. The observed elevations in SII and its correlations with classic inflammatory markers might be attributed to the heightened immune-inflammatory state of children experiencing active JIA, which causes increased proinflammatory signaling and systemic inflammation [70]. A better understanding of these immunological dynamics would provide valuable insights into the complex pathophysiology of active JIA and its potential implications for diagnostic and therapeutic approaches. We employed logistic regression analysis to further characterize the relationship between SII and the diagnosis of JIA and found SII to be the only independent factor associated with the diagnosis of JIA in the study population, even after considering the potential influence of age. The adjustment was necessary since children from smaller age groups normally have more elevated lymphocyte counts [71], resulting in lower NLR and SII values. Therefore, while it can be argued that children from the ReA group were younger than those with JIA (7.7 years, IQR: 3.5, 11.9, and 12.1 years, IQR: 7.6, 14.5, respectively), SII retained significance as an independent factor linked to the diagnosis of JIA after adjusting for age. Furthermore, according to ROC analysis, SII was the only parameter that demonstrated acceptable discriminatory ability (AUC = 0.722), with a higher sensitivity (0.710) but slightly lower specificity (0.541), which indicates a higher rate of false positives. This underlines the need for integrating SII into a diagnostic approach that considers several other aspects, such as the duration of symptoms, family history of autoimmune diseases, and the imagistic aspect of the affected joint. Also, given the relatively low number of patients that can cause variability in estimates, increasing the sample size may enhance the precision of the diagnostic performance measures.

The superior discriminatory performance of SII compared to NLR in identifying active JIA within our study cohort stems from the significant influence of thrombocytosis in active JIA [72]. Children with active JIA had a mean platelet count of 378 × 10^9^/mm^3^ (IQR: 311, 446), while those with Reactive Arthritis had a mean value of 284 × 10^9^/mm^3^ (IQR: 258, 375). Besides their recognized role in coagulation [28], platelets actively coordinate inflammatory responses and immune processes [73,74,75]. Activated platelets possess antigen-presenting properties that enable the immune response of T lymphocytes [75]. Also, they can release proinflammatory microparticles in both peripheral blood and synovial fluid [76]. Concerning Juvenile Idiopathic Arthritis, Güneş et al. described elevated platelet count in children with active JIA, as opposed to those in remission and healthy controls [64]. Liang et al. also observed a significant increase in platelet count in JIA compared to children with Reactive Arthritis [66]. The elevated platelet count in children with JIA compared to those with Reactive Arthritis may reflect the intensity and chronic nature of the inflammatory response associated with JIA [77]. Inflammatory conditions, such as JIA, can stimulate platelet production in the bone marrow and alter their lifespan, leading to an increase in circulating platelet levels [74,78]. The inflammatory milieu in JIA involves various immune cells and cytokines that contribute to the activation of platelets [46]. On the other hand, Reactive Arthritis, often triggered by infections [79], may not induce the same degree of systemic inflammation as JIA. Infections typically lead to a more transient and localized inflammatory response, which may not always result in a significant rise in platelet production [80].

In summary, children experiencing active JIA in our study displayed a significant elevation of SII compared to those with Reactive Arthritis; furthermore, this CBC-derived index was positively correlated with both traditional inflammatory biomarkers, CRP and ESR. This could be additional proof of the inflammatory burden of JIA, and future studies regarding the predictive value of SII regarding disease activity and outcome are warranted.

Nevertheless, it is important to acknowledge some limitations when interpreting these results. First, the retrospective single-center design of the study may have caused selection bias, altering the general applicability of the findings. Second, as the study groups were comprised of real-life patients, some received non-steroidal anti-inflammatory drugs or immunosuppressants at the time of the evaluation, potentially causing some confounding bias. Also, the relatively small sample of patients precludes us from being able to validate specific cutoff values for SII in children with active JIA. Thus, our findings should be considered exploratory. Moving forward, efforts to expand the sample size and validate our findings in larger cohorts will enhance the generalizability and robustness of our results.

## 5. Conclusions

The present study found SII to have moderate diagnostic accuracy in discriminating JIA patients with active disease from arthritis of other causes. Given that the index is derived from the CBC, a readily available, low-cost analysis, it could have complementary value in discriminating JIA patients in cases with negative serology.

## Figures and Tables

**Table 1:** General characteristics across study groups.

Variables	Total (*n* = 108)	JIA (*n* = 70)	ReA (*n* = 38)	R-Value	*p*-Value
Age (years)	10.6 (5.4, 14.3)	12.1 (7.6, 14.5)	7.7 (3.5, 11.9)	0.698	**<0.001**
Females % (*n*)	46.3 (50)	52.9 (37)	34.2 (13)	0.179	0.063
Number of affected joints % (*n*)					
• Oligoarticular	72.2 (78)	67.1 (47)	81.6 (31)	0.154	0.110
• Polyarticular	27.8 (30)	32.9 (23)	18.4 (7)	0.154	0.110
Affected joints % (*n*)					
• Small hand joints	16.7 (18)	21.4 (15)	7.9 (3)	0.173	0.104
• Wrist	12 (13)	15.7 (11)	5.3 (2)	0.153	0.133
• Elbow	7.4 (8)	5.7 (4)	10.5 (4)	−0.088	0.448
• Shoulder	4.6 (5)	7.1 (5)	0	0.162	0.159
• Small feet joints	14.8 (16)	17.1 (12)	10.5 (4)	0.089	0.410
• Ankle	42.6 (46)	47.1 (33)	34.2 (13)	0.125	0.194
• Knee	50 (54)	50 (35)	50 (19)	0	**1**
• Hip	18.5 (15)	11.4 (8)	31.6 (12)	−0.377	**<0.001**
• Spine	10.2 (11)	15.7 (11)	0	0.248	**0.008**
CRP mg/L	11.9 (1.88, 42.8)	13.1 (4.8, 50.6)	7.21 (0.49, 34.9)	0.598	0.093
ESR mm/h	35 (18, 69)	41 (20, 81.2)	24 (10, 47)	0.643	**0.017**
Fibrinogen mg/dL	389 (315.5, 457)	397 (318, 442)	369 (294, 476)	0.5	1
Gamma globulins %	14.8 (11.7, 17.4)	15.6 (12.1, 17.6)	12.5 (11.4, 17.4)	0.611	0.158
IgG g/L	12.3 (10.2, 16.1)	13.13 (10.77, 17.15)	10.55 (7.51, 14.92)	0.633	0.085

Abbreviations: JIA, Juvenile Idiopathic Arthritis; ReA, Reactive Arthritis; CRP, C-reactive protein; ESR, Erythrocyte Sedimentation Rate; IgG, Immunoglobulin G. Statistically significant differences (*p* < 0.05) are represented in bold.

**Table 2:** Comparison of blood cell counts and indices between groups.

Variables	Total (*n* = 108)	JIA (*n* = 70)	ReA (*n* = 38)	R-Value	*p*-Value
WBCs (×10^3^/mm^3^)	8.92 (7.24, 10.73)	8.83 (7.30, 10.69)	8.99 (6.85, 11.26)	0.495	0.946
Neutrophils (×10^3^/mm^3^)	4.87 (3.79, 6.53)	5.16 (4.26, 6.49)	4.20 (2.56, 6.85)	0.616	**0.046**
Lymphocytes (×10^3^/mm^3^)	2.79 (1.97, 3.49)	2.68 (1.84, 3.37)	2.96 (2.39, 3.88)	0.399	0.090
Thrombocytes (×10^9^/mm^3^)	350 (283, 431)	378 (311, 446)	284 (258, 375)	0.693	**<0.001**
Monocytes (×10^3^/mm^3^)	0.770 (0.610, 0.962)	0.740 (0.550, 0.907)	0.840 (0.660, 1.150)	0.366	**0.025**
Eosinophils (×10^3^/mm^3^)	0.130 (0.090, 0.295)	0.120 (0.072, 0.250)	0.200 (0.110, 0.330)	0.382	**0.049**
Hb (g/dL)	11.9 (10.9, 13)	11.8 (10.8, 12.5)	12.3 (11.05, 13.5)	0.415	0.154
RDW (%)	13.2 (12.8, 14.4)	13.5 (12.8, 14.6)	13.1 (12.6, 13.6)	0.585	0.150
NLR	1.82 (1.23, 2.86)	2.12 (1.29, 3.07)	1.33 (0.84, 2.19)	0.667	**0.005**
SII	691 (383, 1071)	779.9 (480, 1233)	410 (266, 737)	0.721	**<0.001**

Abbreviations: JIA, Juvenile Idiopathic Arthritis; ReA, Reactive Arthritis; WBCs, white blood cells; Hb, hemoglobin; RDW, red cell distribution width; NLR, neutrophil-to-lymphocyte ratio; SII, Systemic Immune-Inflammation Index. Statistically significant differences (*p* < 0.05) are represented in bold.

**Table 3:** Binary logistic regression analysis of inflammation factors associated with JIA.

Variables	Univariate Analysis OR (95% CI)	*p*-Value	Variable	Multivariate Analysis OR (95% CI)	*p*-Value
CRP	1.007 (0.997, 1.017)	0.188			
ESR	1.014 (1.001, 1.027)	**0.036**	ESR > 25 mm/h	1.846 (0.763, 4.468)	0.174
NLR	1.313 (0.955, 1.805)	0.094			
SII	1.001 (1.000, 1.002)	**0.005**	SII	1.001 (1.000, 1.002)	**0.037**
Age	1.154 (1.057, 1.260)	**0.001**	Age > 3 years	1.383 (0.356, 5.381)	0.640

Abbreviations: CRP, C-reactive protein; ESR, Erythrocyte Sedimentation Rate; NLR, neutrophil-to-lymphocyte ratio; SII, Systemic Immune-Inflammation Index. Statistically significant differences (*p* < 0.05) are represented in bold.

**Table 4:** Inflammatory Marker Comparison for Discriminating JIA cases.

Variables	AUC	SE	95% CI	Sensitivity	Specificity	Cut-Off	*p*-Value
NLR	0.668	0.058	0.555–0.781	0.667	0.622	1.55	**0.005**
SII	0.722	0.055	0.615–0.829	0.710	0.541	500.9	**<0.001**
CRP	0.599	0.060	0.482–0.716	0.586	0.541	8.92	0.093
ESR	0.644	0.058	0.530–0.757	0.629	0.543	25.5	**0.017**
neutrophils	0.617	0.063	0.493–0.740	0.614	0.579	4.71	**0.046**
lymphocytes	0.600	0.057	0.488–0.712	0.595	0.522	2.76	0.090
platelets	0.693	0.055	0.586–0.801	0.686	0.605	336	**0.001**

Abbreviations: NLR, neutrophil-to-lymphocyte ratio; SII, Systemic Immune-Inflammation Index; CRP, C-reactive protein; ESR, Erythrocyte Sedimentation Rate; AUC, area under the curve; SE, standard error; 95% CI, 95% confidence interval. Statistically significant differences (*p* < 0.05) are represented in bold.

## Data Availability

Data can be made available upon reasonable request due to ethical restrictions.

## Supplementary Materials

The following supporting information can be downloaded at: https://www.mdpi.com/article/10.3390/biomedicines12010065/s1, Table S1: Prevalence of disease subtypes. Table S2: Correlation analysis of CBC-derived indices and parameters across arthritis patients.
