# Supplementary material for: Evaluating the Diagnostic Performance of Systemic Immune-Inflammation Index in Childhood Inflammatory Arthritis: A Focus on Differentiating Juvenile Idiopathic Arthritis from Reactive Arthritis
Source: Biomedicines. 2023 Dec 27;12(1):65. doi: 10.3390/biomedicines12010065 (PMC10812990; doi:10.3390/biomedicines12010065)
Supplement: Supplementary file 1 [file biomedicines-12-00065-s001.zip › Supplementary Table 1.pdf]

Disease	Frequency (percent/number)
JIA	
• enthesitis related arthritis	31.4 (22)
• polyarthritis, RF negative	20 (14)
• polyarthritis, RF positive	8.6 (6)
• systemic arthritis	7.1 (5)
• psoriatic arthritis	5.7 (4)
• oligoarthritis, persistent or extended	27.1 (19)
Reactive Arthritis	
• nasopharyngeal trigger	71.1 (27)
• urogenital trigger	7,9 (3)
• intestinal trigger	21 (8)
