# Supplementary material for: Evaluating the Diagnostic Performance of Systemic Immune-Inflammation Index in Childhood Inflammatory Arthritis: A Focus on Differentiating Juvenile Idiopathic Arthritis from Reactive Arthritis
Source: Biomedicines. 2023 Dec 27;12(1):65. doi: 10.3390/biomedicines12010065 (PMC10812990; doi:10.3390/biomedicines12010065)
Supplement: Supplementary file 1 [file biomedicines-12-00065-s001.zip › Supplementary Table 2.pdf]

**JIA group**					
		**CRP**	**ESR**	**fibrinogen**	**gamma-glob**
NLR	ϱ	0.563	0.228	0.418	0.064
	p	**< 0.01**	0.078	**< 0.04**	0.679
SII	ϱ	0.579	0.430	0.531	0.195
	p	**< 0.01**	**< 0.01**	**< 0.01**	0.206
**ReA group**					
		**CRP**	**ESR**	**fibrinogen**	**gamma-glob**
NLR	ϱ	0.463	0.182	0.242	0.349
	p	**0.040**	0.303	0.243	0.156
SII	ϱ	0.366	0.171	0.331	0.295
	p	**0.028**	0.335	0.106	0.234
